# The functional significance of aberrant cervical counts in sloths: insights from automated exhaustive analysis of cervical range of motion

**DOI:** 10.1098/rspb.2023.1592

**Published:** 2023-11-01

**Authors:** Luisa J. F. Merten, Armita R. Manafzadeh, Eva C. Herbst, Eli Amson, P. Sebastián Tambusso, Patrick Arnold, John A. Nyakatura

**Affiliations:** ^1^ Comparative Zoology, Institute of Biology, Humboldt University of Berlin, Philippstrasse 12/13, 10115 Berlin, Germany; ^2^ Museum für Naturkunde, Leibniz Institute for Evolution and Biodiversity Science, Invalidenstraße 43, 10115 Berlin, Germany; ^3^ Yale Institute for Biospheric Studies, Yale University, New Haven, CT 06520, USA; ^4^ Department of Earth and Planetary Sciences, Yale University, New Haven, CT 06520, USA; ^5^ Department of Mechanical Engineering and Materials Science, Yale University, New Haven, CT 06520, USA; ^6^ Yale Peabody Museum of Natural History, New Haven, CT 06520, USA; ^7^ Palaeontological Institute and Museum, University of Zurich, Karl-Schmid-Strasse 4, 8006 Zurich, Switzerland; ^8^ Department of Health Sciences and Technology, ETH, University of Zurich, Hönggerbergring 64, 8093 Zurich, Switzerland; ^9^ Staatliches Museum für Naturkunde Stuttgart, Rosenstein 1, 70191 Stuttgart, Germany; ^10^ Departamento de Paleontología, Facultad de Ciencias, Universidad de la República, Iguá 4225, 11400 Montevideo, Uruguay; ^11^ Institute for Biochemistry and Biology, University of Potsdam, Karl-Liebknecht-Strasse 24-25, 14476 Potsdam, Germany

**Keywords:** mobility, kinematics, cervical vertebrae, vertebral biomechanics, articular surfaces, zygapophyses

## Abstract

Besides manatees, the suspensory extant ‘tree sloths’ are the only mammals that deviate from a cervical count (CC) of seven vertebrae. They do so in opposite directions in the two living genera (increased versus decreased CC). Aberrant CCs seemingly reflect neck mobility in both genera, suggesting adaptive significance for their head position during suspensory locomotion and especially increased ability for neck torsion in three-toed sloths. We test two hypotheses in a comparative evolutionary framework by assessing three-dimensional intervertebral range of motion (ROM) based on exhaustive automated detection of bone collisions and joint disarticulation while accounting for interacting rotations of roll, yaw and pitch. First, we hypothesize that the increase of CC also increases overall neck mobility compared with mammals with a regular CC, and vice versa. Second, we hypothesize that the anatomy of the intervertebral articulations determines mobility of the neck. The assessment revealed that CC plays only a secondary role in defining ROM since summed torsion (roll) capacity was primarily determined by vertebral anatomy. Our results thus suggest limited neck rotational adaptive significance of the CC aberration in sloths. Further, the study demonstrates the suitability of our automated approach for the comparative assessment of osteological ROM in vertebral series.

## 1. Introduction

Today sloths are represented by two genera: the two-toed sloths *Choloepus* and the three-toed sloths *Bradypus*. Although they diverged approximately 27 Ma [1], the two taxa convergently evolved highly similar ecologies characterized by their suspensory slow arboreal locomotion [2–4]. The two genera also share an extraordinary anatomical characteristic among mammals: besides manatees, extant sloths are the only mammalian lineages that deviate from the highly conserved cervical count (CC) of seven cervical vertebrae (e.g. [5,6]), albeit in opposite ways in the two genera. *Choloepus* tend to decrease the CC, ranging from seven down to just five cervical vertebrae [7]. By contrast, in *Bradypus* the CC is increased and varies from eight to ten, with a count of nine as the most frequent vertebral number [7].

**Figure 1.**
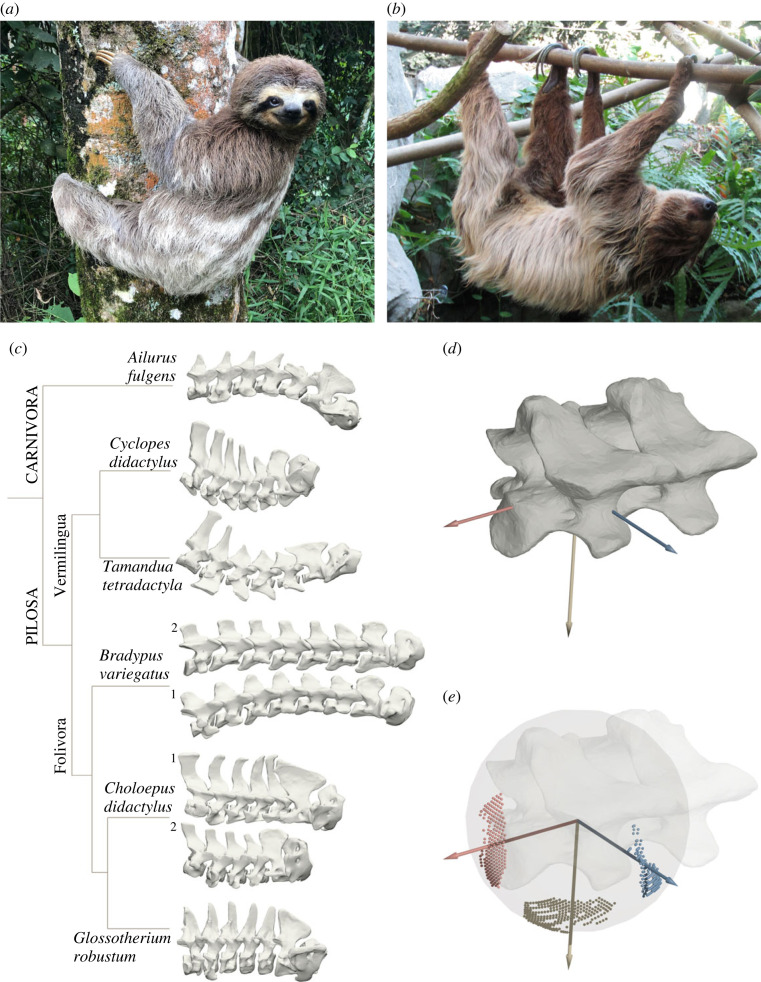
Cervical series and analysis of osteological range of motion. Representative images of (*a*) three-toed sloths (*Bradypus*) and (*b*) two-toed sloths (*Choloepus*). Note the ability of *Bradypus* to turn the neck about the long-axis of the cervical series. Photographs by (*a*) Daniella Maraschiello, distributed under a CC BY-SA 4.0 licence; and (*b*) Fiver der Hellseher, distributed under a CC BY-SA 4.0 licence. (*c*) Phylogenetic relations of the six species represented by the eight individuals. Corresponding three-dimensional reconstruction of the cervical series arranged in ONP (for further explanation, see text) in right lateral view from caudal (left) to cranial (right). Phylogeny based on Presslee *et al*. [1]. (*d,e*) Intervertebral range of motion was measured using anatomically defined coordinate systems. (*d*) Rotations about long-axis (roll, red axis), dorso-ventral axis (yaw, yellow axis) and latero-lateral axis (pitch, blue axis) were modelled as interacting rotations (see text). (*e*) Spherical frame projections (SFPs, see text) help to visualize the interacting rotations of roll, yaw and pitch.

The evolution of decreased or increased CC in *Choloepus* and *Bradypus*, respectively, may be related to the posture and mobility of their heads and necks (figure 1*a*,*b*). *Bradypus* specimens are observed to commonly turn their head around the neck's long-axis so that they can maintain ‘right-side-up’ vision during ‘upside-down’ posture [8]. In contrast, posture of the head and neck of *Choloepus* remains unchanged relative to the rest of the body during suspensory locomotion and posture, causing the animals to see the surroundings ‘upside-down’ [9]. The deviating CC of the two sloth genera in combination with the strikingly high rotatory mobility of the cervical series in *Bradypus* in contrast to a seemingly relatively rigid posture of the cervical series in *Choloepus* suggest functional significance of aberrant CC in both lineages. Therefore, here we test two hypotheses: first, whether the differences of mobility of the cervical column in two-toed and three-toed sloths are a result of the aberrant number of vertebrae, and second, as an alternative but not mutually exclusive explanation for the highly flexible neck of *Bradypus* and rather rigid neck of *Choloepus*, whether specific anatomical features of the vertebrae allow a greater or smaller mobility in the intervertebral joints of the cervical series. We quantitatively test for the presence of such a functional link between aberrant CC and increased or decreased mobility using an automated assessment of the osteological range of motion (ROM) within the cervical series. To allow evaluation of cervical ROM in a broader context, we analyse cervical ROM in a comparative framework covering the Pilosa (i.e. xenarthrans excluding the armadillos) by including an extinct ‘ground sloth’ and two extant anteaters, as well as the red panda as a representative mammal of similar size with a generalized morphology of the cervical series.

Intervertebral movements of mammals involve complex, three-dimensional interactions of several articulations (vertebral bodies and left and right pre- and postzygapophyseal facets; cf. [10]) rendering their *ex vivo* and *in vivo* quantification notoriously challenging (e.g. [11–15]). To date, ROM studies that focused on the cervical series in mammals used different approaches without a consensus on the set-up or analytical technique. For example, Penning & Badoux [16] and Selbie *et al*. [17] used X-ray images of living cats and dogs to quantify movements and determine the intervertebral centre of rotation (COR). Vidal *et al*. [18] and Müller *et al*. [19] used three-dimensional models of the vertebrae of artiodactyls, manually recreated extreme poses, and measured the deflection, e.g. with geometric morphometrics [18]. Another approach that used morphometrics was introduced by Belyeav *et al*. [11,20], in which trigonometric formulae were developed for ROM calculation for various ungulates. Recently, Jones *et al*. [21] provided software to automatically test the ROM for the vertebral joints of a cat for each rotational axis (lateral bending, dorso-ventral flexion and long-axis rotation or torsion of the spine) separately. However, interacting rotations about rotational axes have been qualitatively observed in the mammalian cervical series (e.g. [22–24]) and have been demonstrated experimentally to allow extreme poses of the neck in birds [25,26]. Automated approaches to quantify interacting movements in the less osteologically constrained articulations of the appendicular skeleton, e.g. limb joints, have recently been developed [13,27–32]. Here, we developed this methodology further and employed an automated three-dimensional assessment of intervertebral ROM specifically accounting for interacting rotations of roll, yaw and pitch occurring in the cervical column, using criteria based on both bone–bone collisions and disarticulation. We considered the implications of these results for the adaptive significance of CC of sloths from a functional perspective. Ultimately, we concluded that the shape of the vertebrae—not their count—is largely responsible for the rolling mobility of the cervical series as a whole.

## 2. Material and methods

### (a) Species sampling

The dataset was composed of the cervical series of six species (figure 1*c*). For the extant sloths, the dataset comprised two specimens of *Bradypus variegatus* with a cervical count of eight vertebrae (CC8) each, as well as two specimens of *Choloepus didactylus* with six (CC6) and seven cervical vertebrae (CC7), respectively. We included the extinct ‘ground sloth’ *Glossotherium robustum* as representative of the terrestrial condition more generally found in (extinct) sloths. Although being phylogenetically closer to *Choloepus*, it differs from both genera of extant sloths by its large body size, ground-dwelling ecology and the retention of seven cervical vertebrae (CC7) [33]. The dataset also comprised the two arboreal anteaters *Cyclopes didactylus* (silky anteater) and *Tamandua tetradactyla* (tamandua), as well as *Ailurus fulgens* (red panda), each with seven cervical vertebrae (CC7). The latter was chosen as an outgroup taxon due to its comparable size and habitat but rather generalized anatomy. In the following, we abbreviated the individuals with their genus name (and specimen number for the sloths). The collection number of the specimens can be found in electronic supplementary material, SM0.

### (b) Processing of the three-dimensional bone models

The cervical series ranging from the atlas to the last cervical vertebrae (C1 to C6/7/8) was computed tomography and structured light scanned in different facilities for all specimens (see electronic supplementary material, SM0). Virtual surface models of all vertebrae were then reconstructed in Amira (Version 6.0.0, Thermo Fisher Scientific, Germany) and exported as three-dimensional objects. Meshes were cleaned to ensure manifoldness and mesh count was reduced within the software Geomagic Studio (Version 2013.0.2, 3D Systems GmbH, Germany). Rotations between occiput and C1 and between C1 and C2 were not considered in this study, because C1 anatomy deviates substantially from that of the other cervical vertebrae, which rendered our automated articulation criterion (see below) unsuited for these articulations. All meshes are freely available for download [34].

### (c) Alignment of the cervical series and defining the centre of rotation

Three-dimensional models of each specimen were aligned in the visualization and animation software Maya (Autodesk Maya 2020, Autodesk Inc., USA). The articulated condition of the cervical series was reconstructed by recreating its osteological neutral pose (ONP), which was defined as the position of two adjacent vertebrae when the zygapophyseal facets of the joint were overlapping completely in all the three anatomical planes, i.e. sagittal, frontal and transverse [18]. After the arrangement in ONP (figure 1*c*), the anatomical coordinate system was placed at the centre of rotation (COR, see below), and the spacing of the vertebrae was adjusted to adhere to specific criteria (see electronic supplementary material, SM1). The average relative distance between two vertebral bodies as well as the average relative zygapophyseal distance was calculated based on available X-ray recordings of *Choloepus didactylus* from Nyakatura *et al*. [35] and adjusted to the relative size of the vertebrae for the other species (see electronic supplementary material, SM1, cf. SF1: C + D). Further, given the reported importance of joint translation in mobility studies [30], we assessed sensitivity to joint spacing and intra-observer variability during the orientation of vertebrae in ONP (electronic supplementary material, SM2, SM3, SF1) and found that all general patterns reported in this study hold true irrespective of the unavoidable introduction of subjectivity in these steps.

The position of the COR was approximated by fitting a sphere to the ventral outline of the postzygapophyseal facets of the anterior vertebra in the intervertebral joint, as done previously by Müller *et al*. [19]. Therefore, the COR was set based on postzygapophyseal facet orientation as we expected the potentially interlocking facets to be the motion-limiting components of the joint compared to the relatively simple connection via the intervertebral disc between the vertebral bodies [10,15]. By matching the curvature of the postzygapophyses, the surface of the sphere approximates all hypothetically possible positions of the facets during motion around the spheres' centre (see electronic supplementary material, SF2). The centre of the sphere defined the COR of the Maya ‘joint’ and the position of the anatomical joint coordinate system used for the animation of joint poses (see below); movement about this joint rotated the corresponding anterior vertebra relative to the adjacent posterior vertebra.

### (d) Automated assessment of the range of motion

The analysis of the osteological ROM was based on Maya embedded language (MEL) scripts originally published by Manafzadeh & Padian [31], which were later refined to include a cosine correction [29], and were here extended to accommodate specific constraints of the vertebral movement (see ‘vertBooChecker.mel’; all MEL code used here published by Manafzadeh & Gatesy [13]; freely available for download at https://bitbucket.org/xromm/xromm_other_mel_scripts/src/main/joint_mobility/). A video of the whole workflow can be found in the electronic supplementary material (SM7).

The vertebral joints were hierarchically connected to form a vertebral chain in which the movement of a joint also affected the orientation and position of all cranially articulated (hierarchically ‘down-stream’) vertebrae. Each Maya ‘joint’ and the corresponding coordinate axes were placed and orientated according to the ONP as described above. The hierarchical marionette was built following the Scientific Rotoscoping workflow [5]. Subsequently, the vertebral joints were animated to automatically sample joint poses of interacting rotations along the three movement axes for axial rotation (roll about *x*-axis resulting in rotation of the cervical series' long-axis), lateral bending (yaw about *y*-axis) and sagittal bending (pitch about *z*-axis) with a fixed step size (increment of two rotational degrees for all joints). This method thus represents an exhaustive search of rotational joint pose space (for a recent publication on heuristic ROM analysis, see Bishop *et al*. [27]), but cleaning and downsampling of bone meshes prior to analysis kept computational time tractable (on average approx. 19 000 frames).

Two criteria needed to be met to ultimately qualify a joint pose as viable. First, to test for bone–bone collisions a Boolean intersection operation was run in which Maya calculated the intersection area of the two bone meshes and filtered the intersecting poses to be non-viable (coded ‘0’ for ‘non-viable’). When no intersection mesh was created, the pose was scored as viable (coded ‘1’ for ‘viable’) and was subsequently further tested for articulation. Second, to test for potential disarticulation of zygapophyseal facets, the area of the zygapophyses was duplicated in Maya and then the resulting mesh was moved upwards until it completely occupied the space in-between the articulating facets (cf. electronic supplementary material, SM1), for the left and right zygapophyseal joints. The animated anterior vertebra had to be intersecting with both of these meshes (left and right) to assess whether the zygapophyses were within reasonable distance of each other—i.e. articulated (coded ‘1’)—or disarticulated (coded ‘0’). We decided to rather overestimate the natural ROM of the joints and measured the osteological ROM by defining the disarticulation at 0 percent overlap of the zygapophyseal facets [cf.^21^,^25^,^36^]. Hence the articulation constraints all relied exclusively on osteological features and did not include soft tissue estimations. Through this combination of testing for bone–bone collision and joint disarticulation, the viable poses for the articulated vertebrae could be exclusively selected, and subsequently visualized as spherical frame projections (SFPs; figure 1*d*,*e*) in Maya [37–39] as well as in a three-dimensional cosine-corrected joint pose space [29].

### (e) Visualization of the results

**Figure 2.**
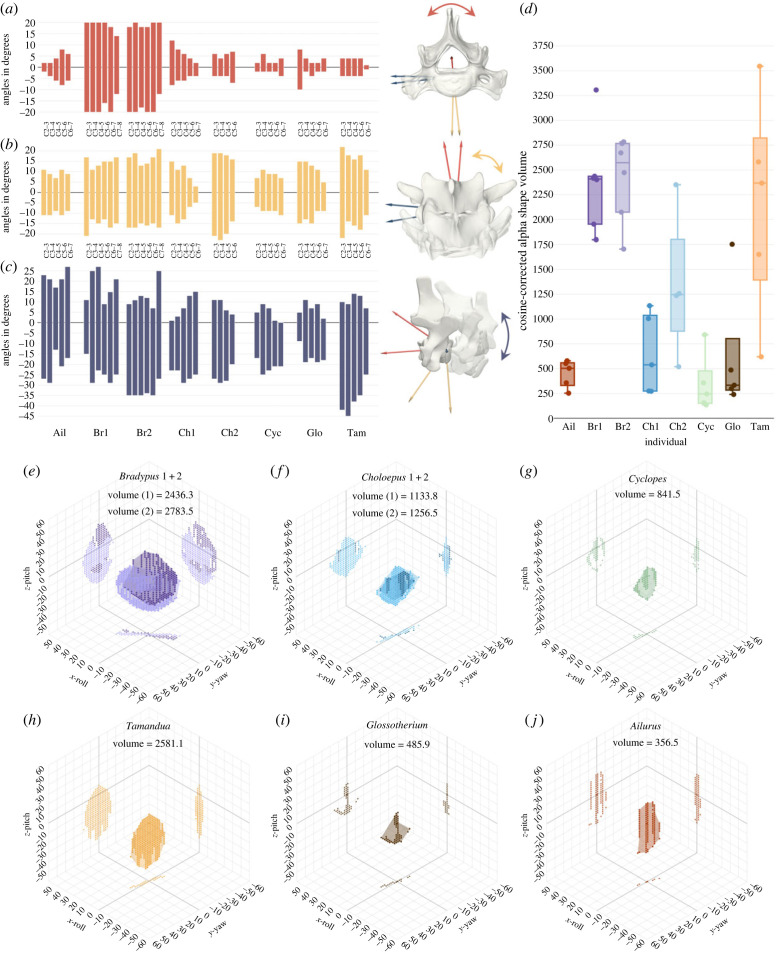
Interactions among rotational degrees of freedom determine ROM within cervical series. (*a*) Maximum roll; (*b*) maximum yaw; (*c*) maximum pitch; each with rendered illustration of the corresponding movements. Deflections of the joints are represented by the bars in ascending order from cranial to caudal (C2 to last cervical). Positive angles indicate clockwise roll (as seen from anterior), yaw to the right and dorsal pitch. (*d*) Intervertebral ROM as assessed by volume (in cubed degrees) of the cosine-corrected alpha shapes. Boxplots for the cervical series of each specimen with corresponding data points for each joint. (*e–j*) Representative alpha shapes for intervertebral ROM of joint C3-4 of each specimen (see electronic supplementary material, SM4, for all joints and SM6 for interactive graphs). Coloured dots for data points hulled by translucent shapes of same colour for alpha shapes. Ail, *Ailurus*; Br1, *Bradypus* 1; Br2, *Bradypus* 2; Ch1, *Choloepus* 1; Ch2, *Choloepus* 2; Cyc, *Cyclopes*; Glo, *Glossotherium*; Tam, *Tamandua*.

A cosine correction of the three-dimensional pose space was conducted for each joint to resolve the inherent distortion of Euler angle space and enable fair quantitative comparison [29]. The results were displayed to illustrate the maximal rotations about each axis, the three-dimensional pose space occupation for each joint of the eight individuals during combined rotation about the three axes, and the volume of the alpha shapes in cubed degrees. An alpha shape is a polytope that hulls a finite set of points in two or three dimensions with an alpha value that determines the level of detail and therefore final shape of the polytope [40]. The viable articulated poses were plotted on an (interactive) three-dimensional graph, the pose space, using the software R and the package ‘plotly’ [41] (figure 2*e–j*; electronic supplementary material, SM4, SM6). The alpha shapes for the pose spaces were created in MatLab (Version R2021a). Either an alpha value of 50 was used or, if the automatically determined critical value was above the preset value of 50, the critical alpha value (in MatLab: the smallest alpha radius that produces an alpha shape that encloses all points) was used. The volume of the alpha shapes was used for comparison of overall mobility among all joints. In addition, the shape of an alpha hull was used to separately compare features of the possible rotations within the pose space (i.e. differences in viable roll, yaw and/or pitch) between the individuals. Therefore, specimens with the same volume of their respective alpha hull could display substantially different shapes, reflecting different partitioning of the same overall three-dimensional mobility.

## 3. Results

### (a) Maximal rotations were symmetrical for roll and yaw, and asymmetrical for pitch in all specimens

The visualization of the minimum and maximum values for the rotation about the three rotational axes during interacting motion of roll, yaw, and pitch demonstrates rather symmetrical distributions for roll and yaw around the ONP, i.e. around the ‘zero pose’ for the joint coordinate system, but a more asymmetrical distribution for pitch (figure 2*a–c*). Within the overall pose space, dorsal pitch was comparably less extensive than ventral pitch for all individuals besides *Ailurus*, for which the maximal pitch angles were on average rather symmetrically distributed around the ONP (figure 2*c*). Roll was generally more restricted than yaw for all individuals besides the two *Bradypus* specimens (figure 2*a*).

The high value for roll in C2-3 of the extinct sloth *Glossotherium* was probably influenced by a damaged right prezygapophysis of the C3 fossil specimen that lacked the lateral bony limitation of an otherwise intact facet. Therefore, we focused our interpretation of the general mobility on its other joints with intact facets.

### (b) Distribution of the poses in three-dimensional pose space and shape and orientation of the alpha hulls

The alpha hulls of the viable, articulated poses for each joint in *Bradypus* (figure 2*e–j*; electronic supplementary material, SM4, SM6) were generally flat, oval, pancake-like shapes that were elongated in *z*-direction (pitch), and oriented diagonally in *x*- and *y*-plane (roll and yaw, respectively). The flat shape of the alpha shapes resulted from a strong interaction of yaw and roll. This implied that specific roll angles were only feasible for certain yaw angles, and vice versa.

The coupling of yaw and roll for both *Bradypus* specimens was almost identical from C2-3 to C7-8, i.e. throughout the whole cervical series, and the line-like outlines of the alpha shapes in *x*–*y* plane were orientated almost perfectly diagonal with an estimated *x*–*y* ratio of approximately 1 (figure 2*e*; electronic supplementary material, SM4: SF3; SM6). Unlike the two *Bradypus* specimens, the two *Choloepus* specimens displayed distinctively different pose spaces in the *x–y* plane (electronic supplementary material, SM4: SF4; SM6). From C2-3 to C6-7 in *Choloepus* 1, the alpha shapes showed a consistent inter-dependency of yaw and roll. By contrast, the step size difference (i.e. the ‘slope’ of the line-like shape in the two-dimensional plane of the graph) of yaw and roll in the cervical series of *Choloepus* 2 was cranially large in C2-3 (e.g. *x* = 4, *y* = 19) but gradually decreased caudally until C5-6 (e.g. *x* = 5, *y* = 10). *Glossotherium*, *Tamandua* and *Cyclopes* showed similar orientations of their alpha hulls throughout the joints with less pronounced *x–y* interaction (i.e. unequal step-sizes of roll and yaw) than in the extant sloth specimens (figure 2*g–i*; electronic supplementary material, SM4: SF5 + 6; SM6). In the cervical series of *Ailurus*, the orientation of the alpha shapes in C2-3 and C3-4 was similar to that of the three last mentioned taxa, whereas the interaction between roll and yaw increased for the caudally following joints (figure 2*j*, electronic supplementary material, SM4, SM6).

The oval alpha shapes further indicated that less rotational mobility for pitch was possible during more extreme angles of roll and yaw (figure 2*e–j*; electronic supplementary material, SM4, SM6). In comparison with each other, the two *Bradypus* specimens showed a great difference in the *z*-extension (pitch) of their viable pose space within the cranial-most joints, which decreased caudally (electronic supplementary material, SM4: SF3; SM6). The z-extension of the alpha shapes for the two *Choloepus* specimens showed some differences between their cervical series. While the viable pitch was considerably larger in the cranial-most joints of *Choloepus* 2, the discrepancy between their joints pitch ability grew smaller caudally until by joint C4-5 the *z*-extensions of their alpha hulls were approximately the same (electronic supplementary material, SM4: SF4; SM6). Within the cervical series of the remaining taxa the alpha shapes also grew smaller caudally in *z*-extension, as observed for the four extant sloths (electronic supplementary material, SM4: SF5 + 6; SM6).

### (c) Summed maximal rotations along each axis for the entire cervical series

The alpha shape volumes indicating the size of the overall viable pose space along the joints in the cervical series of both *Bradypus* specimens (CC8) were on average the largest of all specimens (figure 2*d*). *Tamandua* stood out from the other specimens in showing high variation in alpha shape volume along the cervical series (the functional significance of this result remains unknown). By contrast, all other specimens had a smaller range of values.

**Figure 3.**
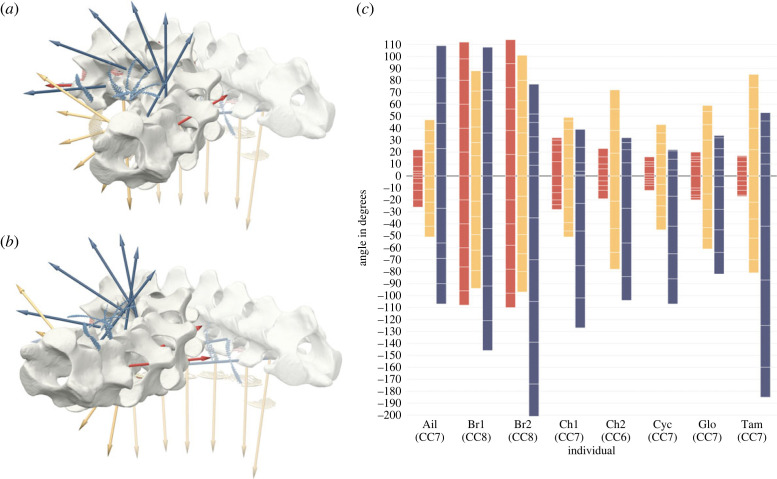
Overall ROM of cervical series. (*a,b*) Superimposed renderings of the cervical series of *Bradypus* 1 arranged in ONP (transparent) and maximum neck-turn to the right in oblique view. For the turn of each joint pose with the maximal deflection for yaw, roll and pitch (priority descending) was chosen. (*a*) Without yaw between C7 and C8 (i.e. simulating a CC of seven), and (*b*) with yaw between C7 and C8. Note that in order to achieve maximum flexion to one side, yaw is strongly interacting with roll. (*c*) Summed ROM for each movement axis including all joints (except for C1-2) for each individual (corresponding CC in parentheses). Roll around *x*-axis (red), yaw around *y*-axis (yellow), and pitch around *z*-axis (blue). White lines within bars part the contributions of each joint to the overall ROM. Positive angles indicate clockwise roll (as seen from anterior), yaw to the animal's right and dorsal pitch. Ail, *Ailurus*; Br1, *Bradypus* 1; Br2, *Bradypus* 2; Ch1, *Choloepus* 1; Ch2, *Choloepus* 2; Cyc, *Cyclopes*; Glo, *Glossotherium*; Tam, *Tamandua.* Note the overall osteological ability to roll of more than 240° in both *Bradypus* specimens.

The maximal viable rotations in each degree of freedom (roll, yaw, pitch) were summed for the entire cervical series of each specimen to evaluate the overall ROM of the cervical vertebral column (figure 3*c*). The visualization of the possible roll about the long-axis of the series demonstrates a symmetry for clockwise and counter-clockwise rotations in all individuals and showed clearly that the two *Bradypus* specimens exhibited the overall greatest ability to rotate their necks about its long-axis. *Bradypus* with the highest CC also had the highest joint-level roll ROM. Notably, the two specimens of *Bradypus* thus did not display a favoured direction of roll.

The patterns were less clear for yaw. All eight specimens exhibited a similar ROM on the level of their individual joints. The two *Bradypus* specimens displayed the highest total yaw rotational angles for the entire cervical series in our sample, followed by *Tamandua* (figures 3*c* and 2*b*). *Choloepus* 2 (CC6) had the fourth largest summed yaw angles and had overall larger ROM for yaw and roll than *Choloepus* 1 (CC7) (figures 2*b* and 3*c*). The extinct sloth *Glossotherium* had ROM values between those of both *Choloepus* specimens.

The summed potential for dorsal and ventral pitch was almost identical in *Ailurus,* whereas for the other specimens summed ventral pitch was more pronounced than summed dorsal pitch. Moreover, ventral and dorsal pitch revealed a difference between both *Bradypus* specimens. *Bradypus* 1 had larger total dorsal, but lower total ventral viable pitch ROM than *Bradypus* 2.

## 4. Discussion

The results of our three-dimensional assessment of cervical ROM allowed us to determine the different features that accompanied the osteological mobility of the neck of two-toed sloths (*Choloepus*) and three-toed sloths (*Bradypus*), as well as of closely related species (*Glossotherium*, *Cyclopes*, *Tamandua*) and a generalized mammal (*Ailurus*). Our automated ROM analysis systematically and exhaustively determined the osteologically viable, articulated poses for each cervical vertebral joint while taking into account interacting rotations about all three joint coordinate system axes, i.e. roll (*x*-axis), yaw (*y*-axis) and pitch (*z*-axis).

Determination of the ONP, the COR, and the articulation constraints all relied exclusively on osteological features in our study. This facilitates the reproducibility of the results, as illustrated by the sensitivity analysis for the intra-observer variation (electronic supplementary material, SM2, SM3). Comparing the ROM of the most generalized specimen in our dataset—*Ailurus*—with the experimental results of the cat from C3-4 to C6-7 from the study of Jones *et al*. [42], and keeping in mind that we measured the osteological ROM which rather overestimates the natural ROM of the joints, the range and overall pattern were indeed similar. The comparability of the values strengthened the point that relying only on bony constraints without incorporating actual soft tissue limitations allowed plausible inter-species comparisons that were still comparable to the condition in living animals. Hence, our study also allowed comparison of extant and extinct species. Thus, the ROM inferred here represents the maximum mobility provided by the bony structures and might have been smaller *in* *vivo* due to the limitations of soft tissues (see discussion of soft tissue effects [18,31,44–48]). By contrast, global interspecific comparisons to gain insight into evolutionary trends of the effects of osteological shape on ROM appear to be a more suitable application of such studies.

### (a) Cervical count does not exclusively govern mobility of the cervical series

We tested two hypotheses related to the functional significance of the CC to gain insight into the striking difference of neck mobility between *Choloepus* and *Bradypus,* specifically the ability for roll within the cervical series (i.e. to rotate the neck about its long-axis) [cf.^7^,^8^]. Our first hypothesis was that the decreased CC in *Choloepus* and increased CC in *Bradypus* result in a decreased and increased mobility of cervical series, respectively. Our second hypothesis was that differences in individual vertebral anatomy result in the observed differences in mobility (see electronic supplementary material, SM5).

Our study highlighted the major importance of diverging vertebral anatomy and showed that not the CC but the shape of the vertebrae was largely responsible for the rolling mobility of the cervical series as a whole. Although *Bradypus* with a CC of eight showed also the highest summed ROM for roll, yaw and pitch along the cervical series, the other specimens with the same CC of seven showed high variations in the ROM and ROM volume among their joints. This resulted in different overall mobilities of the entire cervical series for the same CC (figures 2*a–c* and 3*c*; electronic supplementary material, SM4, SM6). Moreover, *Choloepus* 2 had larger total roll and yaw than *Choloepus* 1, although *Choloepus* 1 had a higher cervical count (figure 3*c*). Taken together, this weakens the hypothesis of a straightforward correlation between CC and overall neck mobility and strengthens the importance of divergent morphology. Additionally, removing 1 or 2 of the eight vertebrae from the cervical series of *Bradypus* would still produce a higher ROM than shown in *Choloepus* (six to seven cervical vertebrae), highlighting the contribution of joint-level mobility to the overall neck ROM.

However, we found that cervical count did play a role in determining overall neck yaw and pitch of *Bradypus*. Only the higher CC of *Bradypus* resulted in an overall larger ROM for yaw and pitch than in *Tamandua*, since the general intervertebral yaw angles between the two were similar or even less for *Bradypus* (figures 2*b*,*c* and 3*c*).

Nevertheless, the ROM analysis and renderings of the cervical series of *Bradypus* 1 for the maximum neck turn (figure 3*a*,*b*) displayed that the extreme roll of their neck did not depend on the contribution of the ‘additional’ joint. This suggests that rolling is the major component of the movement whereas yaw and pitch only play a minor role.

Our results have ramifications for the speculation about the enigmatic breaking of the ‘rule of seven’ exhibited only by extant sloths and manatees among mammals [cf.^6^,^43^,^49^]. Previous studies showed that the divergent CC of *Choloepus* (and potentially also *Bradypus*) is likely the result of a homeotic change caused by shifts in the *Hox* gene expression boundaries in the neck [49], which is usually accompanied by major congenital abnormalities in other mammals [50]. The sloths' low metabolism was proposed to be the key factor that prevented the lethal outcome connected to the aberrant CCs and facilitated neck evolution of both lineages [50,51]. Our results appear to take neck rolling mobility off the list of potential drivers for this evolutionary aberration, despite demonstrated increased overall mobility in regards of yaw and pitch. Alternatively, the aberrant CC might instead be a result of developmental variability, in accordance with intraspecific variability in this trait [50].

### (b) Mobility of the cervical series of two-toed and three-toed sloths

Comparing *Bradypus* to other vertebrates with more than seven cervical vertebrae and a very flexible neck, we find that in addition to the high CC the cervical vertebral column in other species was often divided into sections of varying flexibility [44,52]. Such a functional differentiation of sections has also been proposed for the cervical series of mammals [43]. Krings *et al*. [52] identified a functional division of the cervical series of barn owls (with 14 cervical vertebrae) into several regions of varying flexibility that in combination facilitated head rotations of up to 270°. Although the total of 14 cervicals presumably made this division into six or even seven functionally distinct regions possible, it was the specialized anatomy of individual vertebrae that allowed the large head turns. The neck of ostriches with 15 cervical vertebrae has also been sectioned into three functional segments: a slightly flexible anterior section, a very flexible middle section and a stiff posterior section [44]. This regionalization of the cervical series in birds with at least 14 cervical vertebrae underlined that more cervical vertebrae did not automatically result in a higher flexibility of the neck and that the mobility of the intervertebral joints played a major role. Giraffes, with just seven cervical vertebrae but long, flexible necks, demonstrate that the flexibility of the neck is not necessarily bound to a high CC. Compared to sauropod dinosaurs with a similarly long neck but 12 cervical vertebrae, giraffes had greater osteological ROM per vertebral pair [18]. Together these studies suggest two things. First, in mammals the highly conserved CC of seven led to evolving increased flexibility of the neck only by altering the vertebral anatomy [15,43,53]. Second, in non-mammalian amniotes that were not evolutionarily constrained to a certain CC (i.e. the ‘rule of seven’), alterations of CC as well as alterations in vertebral morphology can increase neck mobility to meet specific functional demands. Sloths represent an interesting case for these two conditions: they are mammals but deviate from the ‘rule of seven’.

The vertebrae of the individuals with the maximum mean possible pitch (*Ailurus*, *Bradypus* and *Tamandua*) displayed two distinctive anatomical features that allowed the greater pitch mobility between their vertebrae (see electronic supplementary material, SM5). The first characteristic were the caudally angled, ventral edges of the anterior and posterior epiphyses of the vertebral bodies. This characteristic shape was the most prominent for the vertebrae of the two *Bradypus* specimens with elongate, conical vertebral bodies in lateral view. The tilted posterior and anterior faces of the vertebral bodies allowed the vertebrae to slide along each other and avoid bone–bone collisions during pitching, reminiscent of the joint motion for vertebrae with more rounded vertebral bodies, e.g. of ruminants and camelids (cf. [19]). This left viable pitch to be limited primarily by the disarticulation of the zygapophyses. In contrast to that*,* the rather square vertebral bodies (in lateral view) of the more slender vertebrae of the remaining species (*Choloepus*, *Cyclopes* and *Glossotherium*) exhibited lower mean maximal pitch mobility. This is consistent with Pierce *et al*. [54], who mentioned that spool-shaped vertebrae, i.e. vertebrae with relatively long vertebral bodies (such as *Ailurus*, *Bradypus* and *Tamandua*), show greater intervertebral flexibility due to the reduction of the contact surface area and the increase in angular deviation between successive vertebrae. By contrast, disc-shaped vertebrae, i.e. vertebrae with short vertebral bodies (such as *Choloepus* and *Glossotherium*), show greater intervertebral rigidity due to a greater contact surface and a minimum degree of deflection before the adjacent vertebrae obstruct each other. The cranio-caudal length of the zygapophyseal facets was the second anatomical feature that facilitated larger viable pitch angles. The long, anteriorly protruding prezygapophyses of the vertebrae of *Ailurus*, *Bradypus* and *Tamandua* elongated the path along which the anteriorly adjacent vertebra could either pitch ventrally before the zygapophyses were no longer overlapping (i.e. were no longer articulated) or pitch dorsally until their postzygapophyses contacted the neural arch and/or spinous process of the posterior vertebra. The elongation of the arc of motion by an increased length of the pre- or postzygapophyses was also observed for Camelidae and Giraffidae [11]. Although other authors stated that long zygapophyses restrict joint mobility, this was often only expected for the rotation about the yaw and roll axes [19,26]. In any case, this aspect merits further investigation, for example using virtually modified (i.e. elongated and/or shortened) zygapophyseal facets in virtual experiments.

Another feature that also influenced ventral pitch in the present ROM analysis was the general shape of the zygapophyses (electronic supplementary material, SM5). In *Choloepus* the angular anterior edges of the prezygapophyses of the posterior vertebra caused an earlier disarticulation during ventral pitch of the two vertebrae of an intervertebral articulation, because the rounded postzygapophyses of the anterior vertebra left less room to overlap with the prezygapophyses of the posterior vertebra. However, this feature is only relevant for the osteological ROM, because in the living animal ligaments and tissues would limit such extensive movements—an aspect that presents an obvious limitation to the assessment of actual mobility in a study of osteological ROM (see above). In the context of an interspecific comparative study design as has been followed here, this limitation is not as critical as long as all specimens are treated in the same way. The cranially gradually decreasing size of the spinous processes in *Cyclopes* and *Tamandua* appears to be precisely adjusted to not interfere during dorsal pitching. C2-3 and to a lesser extent C3-4 of *Choloepus* 1 were the only joints where the contact of the spinous processes of both vertebrae limited further dorsal pitching in the joints. This led to the assumption that in this case the morphology restricts hyper-extension of the neck. A similar condition was found in the lumbar spine of *Choloepus* [14].

### (c) Interactions among rotational degrees of freedom and advantages of our automated analysis approach

Vertebral rotations are not merely excursions about three separate perpendicular axes (i.e. the long-axis, the latero-lateral axis and the dorso-ventral axis). Instead, many poses can only be reached in combined motions, as has previously been demonstrated experimentally for bird necks [25,26]. For example, at a given degree of pitch more long-axis rotation (roll) may be possible than at another degree of pitch, meaning that considering interactions among these rotations is essential to ensure that ROM is neither under- nor overestimated. Our automated analysis of the ROM of vertebral joints is based on the extension of previously published methodology [29,31], which tested the interacting rotations along the three axes for roll, yaw and pitch. The approach therefore accounted for an important aspect not covered by other recent automated analysis techniques (e.g. ‘AutoBend’ by Jones *et al*. [21]), or by the use of trigonometric formulae (e.g. [11,20]). Methods that do account for interacting rotations and automatically assess the pose space have not been applied to the complex case of three-dimensional intervertebral movements before [27,31,37]. Moreover, small irregularities in the meshes of the three-dimensional bone models that cause inaccurate intersections of the bones, as discussed by Jones *et al*. [21], in our case did not cause an incorrect assessment of the osteological ROM. This was because the Maya script sampled all combinations of yaw, pitch, and roll between vertebrae independent of each other, with all viable poses eventually enveloped in a three-dimensional volume. Falsely non-viable poses did not terminate the analysis at that particular point, hence there was no need to incorporate a constraint to allow small amounts of anatomically implausible bony intersections, as applied in ‘AutoBend’ [21]. Moreover, the exhaustive and coupled testing of the entire pose space made the analysis less prone to inaccuracies caused by the initial orientation of the Maya joints because the volume of the ROM envelopes was independent of the ‘zero pose’.

Despite their meristic limitations, functional diversity of the mammalian neck is almost as high as in birds, with numerous head–neck postures adopted during foraging, drinking, grooming, exploration, social interaction and locomotion (see [43] and references therein). The complex arrangement of cervical vertebrae to achieve these postures is thereby governed by their three-point articular geometry, which in turn results in interaction between motions around different axes [25,55]. Although described long before in human and horse [56–58], we here for the first time propose an approach to quantify these interactive motions in automated fashion. Our study showed that roll and yaw were tightly interconnected movements. This interacting motion was previously observed within vertebral joints in *in* *vivo* and in cadaveric studies [e.g.^55^,^59^,^60^], and caused a clockwise roll (viewed from anterior) to be combined with yaw to the right (i.e. in this study both positive angles) and vice versa. The orientation as well as the pancake-like shape of the alpha hulls in the three-dimensional pose space plots indicated the degree of the interaction for the different individuals, which we suggest are determined directly by the morphology of the zygapophyseal facets rather than by artifactual kinematic ‘cross-talk’. During the interacting motion of roll and yaw, roll was primarily limited by the anterior tilt of the facets in combination with the height of the lateral, dorsally pointing ridges of the prezygapophyses (cf. electronic supplementary material, SM1). In *Choloepus* and *Tamandua,* which were species with a comparable coupling and also comparable maximal rotations of roll and yaw, the rough fit of the postzygapophyses (anterior vertebra) into the articulation area of the prezygapophyses (posterior vertebra) allowed a high yaw mobility in the joint.

## 5. Conclusion

This study aimed to illuminate the functional significance of the aberrant number of cervical vertebrae in two-toed sloths (reduced CC) and three-toed sloths (increased CC). Qualitative observations appeared to suggest that especially the increased number of vertebrae of three-toed sloths increase the relative potential for roll about the neck's long-axis, whereas the reduced CC in two-toed sloths results in a relatively immobile neck in terms of roll and dorsal pitch. In general, our quantitative results confirm relatively little and relatively large mobility in two-toed and three-toed sloths, respectively. However, in summary, our observations on the morphology of the vertebrae and the results of the automated osteological ROM analysis in a comparative dataset showed that the shape of the vertebrae—rather than their count—was largely responsible for the rolling mobility of the cervical series as a whole. The shape, tilt and dimension of the zygapophyseal facets governed the degree of mobility in the interacting rotations of roll and yaw.

The evolution of aberrant CC in extant sloths has puzzled zoologists for decades and is still far from being well understood. Although alteration in CC theoretically affects overall mobility of the neck by adding or removing joints, our analyses reveal that neck rolling mobility is not a major driver of cervical elongation/shortening in sloths. By contrast, an adaptive response to upside-down posture of the head during suspensory locomotion is primarily realized in vertebral morphology. Alteration of CC in sloths might therefore be a side product of developmental variability that only secondarily contributes to overall neck mobility in *Bradypus* by increasing the number of elements. Our results further highlight again that, despite their superficial similar morphology adapted to arboreal suspensory locomotion, *Choloepus* and *Bradypus* represent different functional as well as developmental vertebral evolutionary trajectories.

## Data Availability

All meshes are freely available for download [34]. The data are provided in electronic supplementary material [61].
